# Accuracy and Monitoring of Pediatric Early Warning Score (PEWS) Scores Prior to Emergent Pediatric Intensive Care Unit (ICU) Transfer: Retrospective Analysis

**DOI:** 10.2196/25991

**Published:** 2021-02-22

**Authors:** Rebecca L Kowalski, Laura Lee, Michael C Spaeder, J Randall Moorman, Jessica Keim-Malpass

**Affiliations:** 1 School of Medicine University of Virginia Charlottesville, VA United States; 2 School of Nursing University of Virginia Charlottesville, VA United States

**Keywords:** pediatric intensive care unit, cardiorespiratory monitoring, hospital transfer, clinical deterioration, monitoring, ICU, intensive care unit, pediatric, retrospective, detection, deterioration, child, accuracy, cohort

## Abstract

**Background:**

Current approaches to early detection of clinical deterioration in children have relied on intermittent track-and-trigger warning scores such as the Pediatric Early Warning Score (PEWS) that rely on periodic assessment and vital sign entry. There are limited data on the utility of these scores prior to events of decompensation leading to pediatric intensive care unit (PICU) transfer.

**Objective:**

The purpose of our study was to determine the accuracy of recorded PEWS scores, assess clinical reasons for transfer, and describe the monitoring practices prior to PICU transfer involving acute decompensation.

**Methods:**

We conducted a retrospective cohort study of patients ≤21 years of age transferred emergently from the acute care pediatric floor to the PICU due to clinical deterioration over an 8-year period. Clinical charts were abstracted to (1) determine the clinical reason for transfer, (2) quantify the frequency of physiological monitoring prior to transfer, and (3) assess the timing and accuracy of the PEWS scores 24 hours prior to transfer.

**Results:**

During the 8-year period, 72 children and adolescents had an emergent PICU transfer due to clinical deterioration, most often due to acute respiratory distress. Only 35% (25/72) of the sample was on continuous telemetry or pulse oximetry monitoring prior to the transfer event, and 47% (34/72) had at least one incorrectly documented PEWS score in the 24 hours prior to the event, with a score underreporting the actual severity of illness.

**Conclusions:**

This analysis provides support for the routine assessment of clinical deterioration and advocates for more research focused on the use and utility of continuous cardiorespiratory monitoring for patients at risk for emergent transfer.

## Introduction

Events of clinical deterioration leading to emergent pediatric intensive care unit (PICU) transfer can have dire consequences for children [1,2]. Children who have events of clinical deterioration while on the acute care floor can have a 13-fold increased risk of hospital mortality; increased morbidity, and longer ICU and overall hospital lengths of stay (LOS) [1,3-5]. Current approaches to identify children at risk for clinical deterioration on the acute care floor include the use of early warning scoring systems, such as the Pediatric Early Warning Score (PEWS), to offer a “triggering” threshold based on physiological severity of illness parameters leading to escalations in care or the use of rapid response teams [6-10].

Despite the widespread use of PEWS, there has been variability in implementation and standard use. In a retrospective study conducted by Akre and colleagues [7], 85.5% of children with a rapid response team or code event leading to emergent ICU transfer had a PEWS score in the critical range documented many hours (median 11 hours, 36 minutes) prior to the event of interest, suggesting there may be challenges with routine assessments, incomplete observations, lack of standardized scoring between clinicians, establishing situational awareness of changing risk scores, or uncertainty in how to initiate an appropriate proactive clinical action [7,11-14]. Further, children likely deteriorate for many different reasons, and a single score is unlikely to detect them all equally well [15]. These reasons may be why the PEWS score has not been shown to decrease hospital mortality despite its utility in initiating rapid response team intervention [10,16].

Further complicating early warning assessment is the unresolved and debated utility of continuous cardiorespiratory monitoring for children on the acute care pediatric floor [17-19]. One specific exemplar where the guidance on continuous cardiorespiratory monitoring is not clear includes hospitalized children with bronchiolitis who have been recently deescalated from supplemental oxygen. The American Academy of Pediatrics Clinical Practice Guideline for this population suggests that the potential benefits of forgoing continuous respiratory monitoring include shorter LOS, decreased alarm fatigue, and decreased cost, whereas the potential harms include delayed detection of hypoxemia and a delay in appropriate weaning of oxygen. The overall continuous monitoring recommendation for hospitalized children with bronchiolitis in the absence of oxygen therapy is labeled a weak recommendation [20]. When McCulloh and colleagues [21] conducted a randomized controlled trial to assess outcomes associated with intermittent versus continuous pulse oximetry for nonhypoxemic infants admitted for bronchiolitis, they found that there was no difference in LOS or use of therapeutic measures between the 2 groups. Parents of children hospitalized for bronchiolitis perceive that the presence of continuous pulse oximetry monitoring is reassuring [22]. Physiological deterioration can happen to a child between routine 8-hour vital sign assessments, and further refinement on who could benefit the most from continuous cardiorespiratory monitoring for early detection of clinical deterioration is an area of much needed clarification.

There is still much to be learned about how PEWS and continuous cardiorespiratory monitoring are used in routine practice environments. We were also particularly interested in how both PEWS and continuous respiratory monitoring were used prior to clinical deterioration in a sample that required emergent PICU transfer and initiation of therapy escalation (ie, “rough” PICU transfer). The purpose of our study was to (1) determine the clinical reason for emergent transfer, (2) quantify the frequency of physiological monitoring prior to transfer, and (3) assess the values, timing, and accuracy of the PEWS scores 24 hours prior to transfer.

## Methods

We conducted a retrospective cohort study of patients ≤21 years of age transferred emergently from the acute care pediatric floor to the PICU due to clinical deterioration from January 2011 to July 2019 in the University of Virginia Children’s Hospital. Emergent transfers with clinical deterioration were defined as those children or adolescents requiring (1) emergent intubation and mechanical ventilation, (2) initiation of vasopressors, (3) stat transfusion of more than one blood product, or (4) transfer following cardiac arrest on the acute care floor. For all transfers not associated with cardiac arrest criteria (items 1-3), they had to be initiated either prior to transfer or within 12 hours of PICU transfer. Bonafide and colleagues [3] previously developed a clinical deterioration metric using the initiation of mechanical ventilation or vasopressors within 12 hours of transfer. We added the criterion of rapid transfusions to capture deteriorating postsurgical cases and unstable hematology-oncology conditions. Clinical notes and orders were adjudicated for each eligible child to ensure that the PICU transfer was due to clinical deterioration and not for planned procedures or postoperative transfers. Children and adolescents also had to be admitted to the acute care floor long enough to have routine care established (at least 6 hours).

To determine the indication for transfer, all available notes for the admission of interest were reviewed by RLK following adjudication definitions used by Blackwell and colleagues [15]. Reasons for clinical deterioration included respiratory distress (leading to emergent intubation or mechanical ventilation), concern for or worsening infection, bleeding or anemia requiring transfusion, cardiac arrest, seizure, stroke, unplanned surgery, or other reasons. These categories were not mutually exclusive, and a child or adolescent could have more than 1 reason for the transfer. Determination of physiological monitoring status was obtained by reviewing the order sets (a medical order for continuous telemetry or pulse oximetry monitoring) prior to the time of transfer to determine if the child or adolescent had an order for continuous telemetry or pulse oximetry monitoring or intermittent vital sign monitoring (every 1, 2, 4, or 8 hours).

Finally, the 3 PEWS scores documented in the electronic medical record (EMR) prior to transfer were abstracted. The University of Virginia implemented a modified PEWS score (Table 1) beginning in 2012 as a part of routine clinical care with the expectation that it was to be completed at every routine nursing assessment and vital sign acquisition. The modified PEWS score closely resembles the Monaghan PEWS score [6] and the automated PEWS (AutoPEWS) score that has been tested for integration within EMR [23]. To determine accuracy, we used the time-concordant heart rate and respiratory rate to determine if any of the categories were underscored or overscored. Of note, capillary refill could not be reliably adjudicated so the scores were only compared for correctness with the available vital sign parameters. Other clinical variables abstracted include age and overall LOS. Descriptive statistics were calculated using R (2019; R Core Team, Vienna, Austria).

**Table 1:** Modified Pediatric Early Warning Score (PEWS).

Category	Score			
	0	1	2	3
Behavior	Playing, appropriate, at patient’s baseline	Inappropriately sleepy *or* fussy but consolable	Irritable *or* inconsolable	Lethargic/confused *or* reduced response to pain
Cardiovascular	Pink *or* capillary refill <2 seconds	Pale *or* capillary refill 3-4 seconds *or* mild tachycardia *or* single ventricle shunted (BT^a^ shunt or Norwood/Sano)	Gray/dusky *or* capillary refill 4-5 seconds *or* moderate tachycardia	Gray/mottled *or* capillary refill >5 seconds *or* severe tachycardia *or* new onset bradycardia
Respiratory	Within normal parameters, no retractions	Mild tachypnea *or* using accessory muscles *or* >30% FiO_2_ or >0.5 L/min/kg	Moderate tachypnea *or* retractions *or* >40% FiO_2_ or >1 L/min/kg	Severe tachypnea *or* RR^b^ < normal for age *or* >50% FiO_2_ or >1.5 L/min/kg

^a^BT: Blalock-Taussig.

^b^RR: respiratory rate.

## Results

We found 72 cases of emergent PICU transfer due to clinical deterioration. The median age was 2.3 years (25% 7.6 months, 75% 11.4 years), and the majority of the children were less than 12 months of age.

The median LOS on the acute care floor prior to transfer was 1.4 days (25% 0.5 days, 75% 2.8 days); 31 children and adolescents (31/72, 43%) transferred within 24 hours of arrival, and 44 (44/72, 66%) transferred within 48 hours of arrival to the acute care floor. The children were severely ill at the time of transfer. Within 6 hours of transfer to the PICU, 54 (54/72, 75%) of patients were emergently intubated, 15 (15/72, 21%) were rapidly transfused, 9 (9/72, 13%) were given vasopressors, and 1 (1/72, 1%) patient experienced a cardiac arrest while on the acute care floor.

Respiratory distress was the most common indication for transfer (36/72, 50%), followed by infection (28/72, 39%), bleeding or anemia requiring transfusion (8/72, 11%), uncontrolled seizure (4/72, 6%), stroke (2/72, 3%), unplanned surgery (2/72, 3%), and other reasons (14/72,19%). Prior to the PICU transfer, only 25 (25/72, 35%) of patients were continuously monitored; 33 (33/72 46%) of the patients had vital signs ordered every 4 hours, 10 (10/72, 14%) had vital signs ordered every 8 hours, and 3 (3/72, 4%) had vital signs ordered every 1 or 2 hours. The overall LOS was long, and the median time in the hospital was 24 days for the sample (25% 10.8, 75% 45.8 days). The mortality of children who were emergently transferred was high (15/72, 21%).

Only 56 of the 72 patients (78%) had documented PEWS scores prior to emergent PICU transfer, and the mean time of the last PEWS score documented prior to transfer was 3.0 hours (SD 3.2 hours). Patients who were not being continuously monitored had higher documented PEWS scores across all 3 time points. In the last recorded PEWS score prior to transfer, those in the group that were continuously monitored had nearly a full point lower average PEWS score (mean 2.2, SD 2.4) compared to those who were not being continuously monitored (mean 3.2, SD 2.2), indicating an increased severity of illness prior to transfer in the noncontinuously monitored group (*P*=.15). There were no clinically nor statistically significant differences in the last recorded PEWS score prior to PICU transfer between children who died and those who did not.

**Figure 1:**
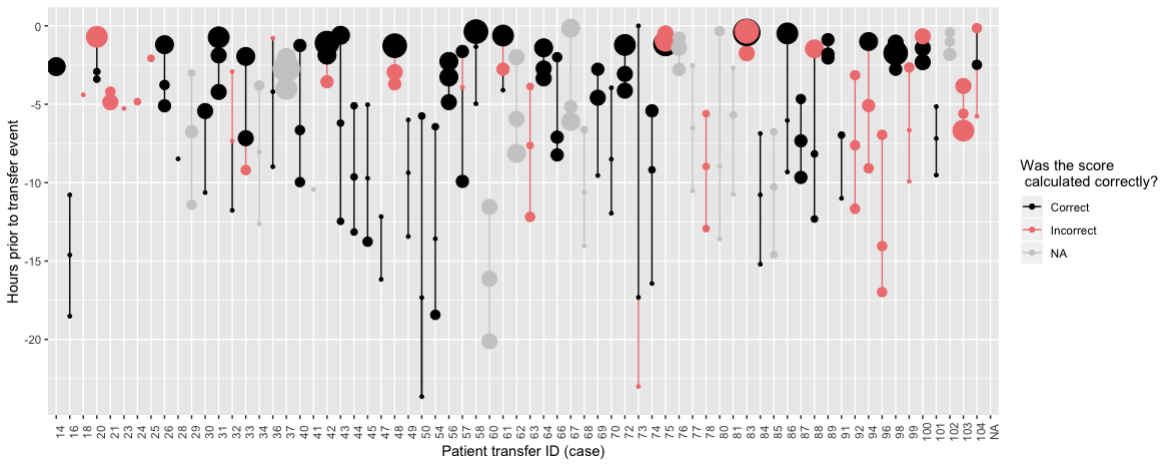
Pediatric Early Warning Score (PEWS) scores prior to emergent transfer. The ordered sequence of PEWS scores in the electronic medical record prior to PICU transfer are shown, indexed in the order they occurred, and the size of the data points is proportional to the score itself. Correct: score was recorded and accurate; Incorrect: score was recorded, but lower than the recalculated value; N/A: score was not recorded.

Figure 1 shows several elements relating to the 3 PEWS scores before transfer. The major finding is that nearly half of the sample (26/72, 47%) had at least 1 incorrectly recorded score in the 24 hours prior to emergent PICU transfer, and all of the errors were underscored PEWS values, meaning that the recorded score in the EMR was less than what it should have been if calculated accurately.

## Discussion

This analysis presents a description of the accuracy of the documented PEWS score and continuous monitoring presence prior to events of clinical deterioration. Early warning scores like PEWS intend to enable clinicians to act early in recognizing clinical deterioration in children. Faced with an already complex workflow, clinicians need to be able to systematically calculate accurate scores, trust the scores, and develop standard practices for proactive care [11,24]. Some of that trust will lie in their availability, accuracy, and ability to determine trends over time. In this retrospective review of emergent PICU transfers, we found that more than 20% of cases had no PEWS recorded, and nearly half of those recorded were underscored, thereby underestimating the actual risk of the child for deterioration. Our finding was similar to the work of Chapman and colleagues [14] who found that only 36% of their sample had adequate vital signs documented to calculate a PEWS score, and when documented, nearly 20% of the PEWS scores contained an error. In their sample, underscoring was more common than overscoring, and 9% of the inaccuracies were deemed clinically significant [14]. Further, when Trubey and colleagues [25] conducted a systematic review of the validity and effectiveness of pediatric early warning systems, they found that the completeness of documentation and interrater reliability of the score varied widely, with some studies only achieving 67% agreement. While an evaluation of the differential accuracy of higher PEWS scores versus lower PEWS scores has not been delineated, it may be that higher respiratory and heart rates (thus higher PEWS scores) may have greater variability in accuracy. This is an area of needed further inquiry.

In addition to the incomplete documentation and inaccuracies in reporting, we found that the PEWS scores were often documented many hours prior to the actual PICU transfer event, indicating incomplete assessments in the hours immediately preceding the transfer when the scores could have been the most helpful in providing early warning of clinical deterioration. Further, the majority of children demonstrated clinical deterioration within 48 hours of arrival to the acute care floor. This finding emphasizes the known challenges with prognostication, defining clinical acuity, and determining the appropriate level of care [26,27]. We found many clinical reasons for deterioration, supporting the notion that there is unlikely to be a single early warning score that adequately captures all types of decompensation [15]. Further, there is substantial heterogeneity in ages represented in any pediatric sample. When Spaeder and colleagues [28] developed a machine learning model to predict early onset of pediatric sepsis, they found that parameters performed differently in the model given the age of the pediatric patient, again indicating that no one model likely performs equally well in all age ranges represented in pediatric care (neonate, infant, child, adolescent).

We note that very few of our patients were continuously monitored with telemetry or pulse oxygenation prior to their emergent PICU transfer. Additionally, those without a continuous monitoring order had higher recorded PEWS scores prior to transfer than those with continuous monitoring, indicating that clinicians may be missing important changes in the underlying physiology when relying on intermittent vital sign assessment alone. Continuous monitoring in children can be challenging to implement because it can be difficult to keep continuous monitoring leads and probes on mobile children, and previous estimates have demonstrated as few as 1% of alarms in children are clinically meaningful [29]. We speculate that there can be clinical benefit to shifting the clinical monitoring paradigm away from its use only as a means of responding to critical physiological alarms and towards a means for early detection of clinical deterioration using continuous predictive analytics monitoring so clinicians can initiate proactive clinical actions [28,30,31]. To avoid medical overuse and further contribution to false alarms, there is a defined need to determine the populations that could benefit the most from continuous cardiorespiratory monitoring in the acute care pediatric setting while also determining the correct “dose” of continuous cardiorespiratory monitoring for those at risk of clinical deterioration.

PEWS, like all point scores of its kind including the Pediatric Rothman Index [32], takes snapshots of clinical status at the time of nurse vital sign assessments. Much can happen clinically between these intermittent events—here, there is a role for continuous physiological monitoring as a means for detecting clinical deterioration. In adults at risk for ICU transfer, metrics extracted from advanced mathematical analyses of monitoring data added information to vital signs and lab tests in early detection of clinical deterioration [31]. Further, continuous predictive analytics monitoring does not rely on arbitrary thresholds of risk cutoff and can incorporate small changes in vital signs, electrocardiogram changes, and laboratory findings, which may cumulatively present a different and more accurate representation of overall risk and represent various clinical etiologies for decompensation [15,33,34]. 

There are several limitations of this analysis that must be noted. Data collection was limited to 1 tertiary academic children’s hospital with a high proportion of children with medical complexity, including complex cardiac surgical cases; therefore, the results may not be generalizable to other settings. Additionally, the sample size was small because we chose a strict classification of emergent PICU transfer with clinical deterioration. Finally, the clinical abstraction was limited to what was available as documentation in the medical record, and there were many instances of a lack of documentation of the PEWS score. Further, there may be changes in continuous monitoring practices without documentation of a written order based on clinical severity at the time of presentation.

This analysis provides support for the routine assessment of clinical deterioration and advocates to extend current monitoring paradigms with the development of continuous predictive analytics monitoring for patients at risk of clinical instability and emergent transfer. It also suggests that more study is needed to determine “who, when, and how much” continuous telemetry or pulse oximetry monitoring should be used and may be the most beneficial for higher risk children and adolescents.

## Abbreviations

EMR: electronic medical record
LOS: length of stay
PEWS: Pediatric Early Warning Score
PICU: pediatric intensive care unit
